# Traumatic Severity and Trait Resilience as Predictors of Posttraumatic Stress Disorder and Depressive Symptoms among Adolescent Survivors of the Wenchuan Earthquake

**DOI:** 10.1371/journal.pone.0089401

**Published:** 2014-02-26

**Authors:** Liuhua Ying, Xinchun Wu, Chongde Lin, Lina Jiang

**Affiliations:** 1 Department of Psychology, Zhejiang Sci-Tech University, Hangzhou, People’s Republic of China; 2 Institute of Developmental Psychology, Beijing Normal University, Beijing, People’s Republic of China; 3 School of Psychology, Beijing Normal University, Beijing, People’s Republic of China; 4 College of Humanities and Law, Hangzhou Dianzi University, Hangzhou, People’s Republic of China; University of Stellenbosch, South Africa

## Abstract

**Purpose:**

To examine the associations between trauma severity, trait resilience, and posttraumatic stress disorder (PTSD) and depressive symptoms among adolescent survivors of the Wenchuan earthquake, China.

**Methods:**

788 participants were randomly selected from secondary schools in the counties of Wenchuan and Maoxian, the two areas most severely affected by the earthquake. Participants completed four main questionnaires including the Child PTSD Symptom Scale, the Center for Epidemiologic Studies Depression Scale for Children, the Connor and Davidson’s Resilience Scale, and the Severity of Exposure to Earthquake Scale.

**Results:**

After adjusting for the effect of age and gender, four aspects of trauma severity (i.e., direct exposure, indirect exposure, worry about others, and house damage) were positively associated with the severity of PTSD and depressive symptoms, whereas trait resilience was negatively associated with PTSD and depressive symptoms and moderated the relationship between subjective experience (i.e., worry about others) and PTSD and depressive symptoms.

**Conclusions:**

Several aspects (i.e., direct exposure, indirect exposure, worry about others, and house damage) of earthquake experiences may be important risk factors for the development and maintenance of PTSD and depression. Additionally, trait resilience exhibits the beneficial impact on PTSD and depressive symptoms and buffers the effect of subjective experience (i.e., worry about others) on PTSD and depressive symptoms.

## Introduction

The 2008 Wenchuan earthquake was the most devastating natural disaster that had occurred in China since the 1976 Tangshan earthquake: An estimated 69, 277 people lost their lives and countless others were injured, displaced, or incurred financial losses. Meanwhile, the earthquake led to a range of negative psychological consequences among child and adult survivors, such as posttraumatic stress disorder, anxiety, depression, and suicidality. Posttraumatic stress disorder (PTSD) is usually considered to be the most prevalent psychopathology in adolescents exposed to the deadly earthquake [1]–[5]. For example, Giannopoulou et al. showed that the prevalence rate of PTSD 6–7 months after the 1999 Athens earthquake was 35.7% among youths aged 9–17 years [4]. In addition to PTSD symptoms, child exposed to traumatic events usually experience comorbid depression. For example, previous studies reported that the prevalence rates of depression ranged from 13.6% to 40.8% in children exposed to an earthquake [1], [6]–[8]. The disparity in rates of PTSD and depression across studies could be attributed differences in the severity of traumatic events, the timing of psychiatric assessment, and the diversity of the research methodologies employed [9], [10].

Aspects of the disaster and disaster exposure are known to impact trauma symptoms. Accruing empirical evidence indicated that increased PTSD and depressive symptoms is related to objective elements of individual trauma experience such as witnessing the disaster, death/injuries of family members, and house damage [11]–[15]. Those individuals with greater objective exposure usually have higher levels of PTSD and general psychopathology. For instance, Thienkura et al. [16] showed that adolescents who lost family members had more severe PTSD symptoms than adolescents without experiencing bereavement. Another study of 2, 250 adolescents (*M*_age_ = 14.6 years, *SD* = 1.3) exposed to the Wenchuan earthquake found that directly witnessing to the trauma event was related to increased risk for PTSD symptoms [1]. Additionally, several studies showed that PTSD and depression were also related to severe house damage [17], [18].

Apart from objective elements of traumatic events, individual’s subjective experiences (e. g., the perception of threat and fear) play an important role in determining posttraumatic response [9], [13], [19]. Moreover, compared to objective aspects (i.e., injury, house damage, proximity to the epicenter) of trauma severity, perceived threat to safety can explain more variances in PTSD symptoms [5]. For example, in a study of 530 adult earthquake survivors (*M*_age_ = 41.32 years, *SD* = 16.36) in Turkey, Basolgu et al. (2004) showed that fear during the earthquake was the most important predictor for the severity of PTSD and depression, explaining the greatest variance in symptoms among all predictor variables (e.g., age, gender, loss of family members, and damage to home) [20].

However, traumatic experiences do not necessarily lead to the development of psychopathological symptoms. A growing number of studies suggest that a considerable proportion of children show no pathology, despite suffering severe adversity that would be expected to produce serious sequelae [21]–[23]. As such, in recent years, considerable attention is now paid to individual resilience following trauma. Although no universal definition of resilience has yet been established, resilience is frequently defined on the basis of two key concepts: adversity and positive adaptation [24], [25]. Conceptually, an important debate diverges on whether resilience should be conceptualized as either a personality trait or a process [24], [26]. When resilience has been conceived as a trait, it usually represents a constellation of characteristics that enable individuals to adapt to the circumstances they encounter, such as optimism, hardiness, strong self-esteem, and social problem solving skills [27], [28]. When resilience has been framed as a process that changes over time [29], it is usually referred to as a “dynamic process encompassing positive adaptation within the context of significant adversity (p. 543)” [30]. For the current research we defined resilience as a cluster of personality traits which are measured by the Connor-Davidson Resilience Scale [28].

These characteristics of resilience enable individuals to deal effectively with the adversity. There is substantial evidence that resilience might help to improve one’s well-being and promote recovery from stressful situations. For example, Catalano and his colleagues [31] found that characteristics of resilience (e.g., tenacity, personal strength, and optimism) can attenuate depressive symptoms among individuals with spinal cord injury living in the community. Additionally, a study of 500 college students (*M*_age_ = 16.7 years, *SD* = 1.2) exposed to a diverse history of trauma showed that higher trait resilience was negatively associated with PTSD symptoms [32]. Furthermore, resilient individuals often exert some personality characteristics that moderate the deleterious effects of stress on healthy outcome. For instance, in a recent study of 1221 German adolescents (*M*_age_ = 24.7 years, *SD* = 2.76), Pinquart (2009) found that the effect of the frequency of daily hassles on concurrent levels of symptoms distress was buffered by dispositional resilience [33].

Based on the literature, the current study examined the associations between traumatic severity, trait resilience, and PTSD as well as depressive symptoms among adolescent survivors of the Wenchuan earthquake. Specifically, we hypothesized that: 1) each aspect of trauma severity (i.e., direct exposure, indirect exposure, worry about others, and house damage) would be positively correlated with PTSD and depressive symptoms; 2) trait resilience would be negatively associated with PTSD and depressive symptoms; and 3) the associations between traumatic severity and PTSD and depressive symptoms would be moderated by individual trait resilience.

## Methods

### Participants and Procedure

Data in the present study were collected as part of an extensive longitudinal study on psychological adjustment among child survivors of the Wenchuan earthquake. In the study, 3, 052 child survivors were randomly selected from 20 primary and secondary schools in the counties of Wenchuan and Maoxian, the two areas most severely affected by the earthquake. These participants, on average, were 13.31 years of age (*SD* = 2.27), with a range from 8 to 19 years old, and 53.5% were female. Four assessments were completed at 12, 18, 24 and 30 months after the Wenchuan earthquake. This project was approved by the local education authorities (i.e., County Departments of Education) and the Research Ethics Committee of Beijing Normal University. Written informed consent was obtained from school principals and classroom teachers. In China, research projects that are approved by local education authorities such as county departments of education and the school administrators and that are deemed to provide a service to the students do not require parental consent. The current project belonged to that category and was thus not required to obtain written informed consent from parents. Students were provided with a description of the research being conducted and were informed that participation was voluntary and they had a right to decline to participate in the study. Written informed consent was obtained from each subject. Under the supervision of trained individuals with a Master’s degree in psychology, participants took about an hour to complete the confidential questionnaires in their classroom. Given that this study was initiated partly to help children cope with the aftermath of the earthquake, no incentives were offered to the students for their participation other than possible counseling if needed.

Of 3052 participants, 2264 participants were not administered intentionally the Connor and Davidson’s Resilience Scale due to the overall length of the study and the appropriateness of measures for different age groups. Thus, the current study analyzed the data from 788 adolescent survivors (54% female) who completed all four main measures: the Child PTSD Symptom Scale (CPSS) [34], the Center for Epidemiologic Studies Depression Scale for Children (CES-DC) [35], [36], the Connor and Davidson’s Resilience Scale (CD-RISC) [28], and the Severity of Exposure to Earthquake Scale. Their age ranged from 12–19 years old (*M* = 15.03, *SD* = 1.65 years).

### Measures

#### Posttraumatic stress disorder

Posttraumatic stress symptom level was assessed with the CPSS [34]. This 17-item self-report measure was designed to assess the severity of DSM-IV-defined PTSD symptoms in relation to the most distressing event. All items were modified so that they were answered in reference to the Wenchuan earthquake the participants recently experienced (e.g. “feeling upset when you think or hear about this earthquake”). Children reported the presence and frequency of symptoms during the past two weeks on a 4-point Likert-type scale, ranging from 0 (*not at all/only at one time*) to 3 (*many times a week or almost always*). Total possible CPSS scores range from 0 to 51, with higher scores indicating greater severity of PTSD symptoms. The original CPSS has demonstrated good psychometric properties [34]. Reliability and validity of the Chinese version of the CPSS has been established [37]–[39]. The Cronbach’s α of the scale in the current study was.89.

#### Depression

Children’s depressive symptoms were assessed using the CES-DC [35], [36]. The CES-DC is a 20-item self-report measure which is designed to assess individual emotional, cognitive, and behavior-related symptoms of depression. Participants indicated how often they fell this way during the past week ranging from 0 (not at all) to 3 (a lot). Thus, total possible scores range from 0 to 60, with higher CES-DC scores be indicative of increasing levels of depressive symptoms. The CES-DC has demonstrated good psychometric properties [40]. The Chinese version of the CES-DC has also been found to have good reliability and construct validity among Chinese populations [41], [42]. The Cronbach’s α of the scale in the present study was.85.

#### Severity of exposure to earthquake

The severity of exposure to the earthquake was assessed with the earthquake exposure questionnaire, which was modified from scales used in prior studies of natural disasters [43], [44]. It consisted of the following items: a) *Survivor’s direct exposure* (2 items): was the participant trapped or injured in the earthquake? (*no* or *yes*); b) *Survivor’s indirect exposure* (22 items): did the participant have a parent, other relative, teacher, classmate, friend, or other person he/she knew who was trapped, injured or died during the earthquake (*none*, *hearing*, or *witnessing*); c) *Worry about others* (8 items): was the participant worried about parents, teachers, classmates or himself/herself dying or being injured during the earthquake? (*no* or *yes*); d) *House damage* (2 items): what was the impact of earthquake on their house and school building? (*none*, *mild*, *severe*, or *totally collapsed*).

#### Trait resilience

Trait resilience was assessed using the Chinese version [35] of CD-RISC [28], a self-report instrument to measure the ability to cope with stress and adversity. The original CD-RISC consists of 25 items, and each item is rated on 5-point likert scale ranging from 0 (not true at all) to 4 (true nearly all of the time). Higher total scores reflected high levels of resilience. A preliminary study of its psychometric properties in general population and patient samples have showed good internal consistency, and construct, convergent, and discriminated validity [28]. Although subsequent studies were not able to replicate the 5-factors structure originally reported [45]–[47], the CD-RISC is still regarded as one of the resilience measures having the best psychometric properties in a meta-analysis [48]. The Chinese version of the CD-RISC was first translated and used by Yu and Zhang (2007) study of Chinese adults [46]. It has good psychometric properties. However, due to the culture differences between the West and the East (e.g., less religious beliefs in Chinese people), the 3-factor model (tenacity, strength, and optimism), not the 5-factor structure, was found in their study [46]. Thus, with consideration of the instability of factor structure, we only used the total scores in the current study. The Cronbach’s α of the scale in the present study was.93.

## Results

### Descriptive Statistics and Intercorrelations among Main Variables

Based on the DSM-IV [49], subjects were identified as having full PTSD according to the following criteria: (a) one or more items of the intrusion subscale scored 2 or 3; (b) three or more items of the avoidance subscale scored 2 or 3; (c) two or more items of the hyper-arousal subscale scored 2 or 3. According to these criteria, the prevalence rate of *probable* PTSD was 12.8% (*n* = 101). In addition, Weissman et al. [50], who were the developers of the CES-DC, have used the cutoff score of 15 as being suggestive of depressive symptoms in children and adolescents. According to that criterion, the prevalence rate of *probable* depression was 51.3% (*n* = 404). Among those participants with *probable* PTSD, the prevalence rate of comorbidity between *probable* PTSD and depression was 98% (*n* = 99).

Means, standard deviations, zero-order correlations of all variables were presented in Table 1. As expected, trait resilience had significant and negative correlations with adolescent PTSD (*r* = −.11) and depressive and depressive symptoms (*r* = −.19). In contrast, each aspect (i.e., direct exposure, indirect exposure, worry about others, and house damage) of trauma severity was positively and significantly correlated with PTSD and depressive symptoms, with correlation coefficients ranging from.09 to.25. In addition, compared to male participants, female participants had more severe symptoms of PTSD and depression.

**Table 1:** Descriptive statistics and intercorrelation among main variables.

	*Mean*	*SD*	1	2	3	4	5	6	7	8	9
1. Age	15.03	1.64	–								
2. Gender^a^	–	–	.03	–							
3. Direct Exposure	.16	.26	.12**	.02	–						
4. Indirect Exposure	.37	.31	.09**	–.04	.39**	–					
5. Worry about Others	.76	.30	.02	–.19**	.07	.21**	–				
6. House damage	2.07	.57	.10**	–.03	.17**	.41**	.10**	–			
7. Resilience	2.20	.70	.16**	–.02	.06	.07	.05	.02	–		
8. PTSD symptoms	.93	.50	.06	–.22**	.10**	.25**	.21**	.14**	–.11**	–	
9. Depressive symptoms	1.12	.48	.15**	–.17**	.09**	.19**	.17**	.10**	–.19**	.78**	–

Note. PTSD = Posttraumatic stress disorder;

a for adolescent participants’ gender, 0 =  female, 1 =  male;

**p<.01.

### Hierarchical Multiple Regression Analyses

To examine the effect of trauma severity and trait resilience on PTSD and depressive symptoms, we conducted a series of hierarchical regression analyses following the same procedure each time. In these analyses, the dependent variables were PTSD and depressive symptoms. Independent variables of each regression analysis included control variables (age and gender), one of the four indicators of trauma severity (e.g., house damage), trait resilience, and the interaction term involving trauma severity measure and trait resilience. All independent variables were centered on their respective means to reduce the multicollinearity between main effects and the interaction term and to increase the ability of β weights for interaction terms [51].

As showed in Table 2, after controlling the effect of age and gender, each aspect of trauma severity (i.e., direct exposure, indirect exposure, worry about others, and house damage) were positively and significantly related to individual PTSD and depressive symptoms, whereas trait resilience was negatively and significantly associated with individual PTSD and depressive symptoms. In addition, the interaction between worry about others and trait resilience was significantly related to PTSD and depressive symptoms.

**Table 2:** Trauma severity and trait resilience as predictors of PTSD and depressive symptoms among adolescent survivors of the Wenchuan earthquake.

Variables	PTSD		Depression	
	β	Δ*R^2^*	β	Δ*R^2^*
Step 1: Control variables		.05		.05
Age	.07		.15***	
Gender^a^	–.22***		–.18***	
Step 2: Independentvariables		.03		.06
Direct exposure	.11***		.08*	
Resilience	–.13***		–.23***	
Step 3: Interaction		.00		.00
Direct exposure ×Resilience	–.01		–.03	
Step 1: Control variables		.05		.05
Age	.07		.15***	
Gender^a^	–.22***		–.18***	
Step 2: Independentvariables		.08		.08
Indirect exposure	.25***		.18***	
Resilience	–.14***		–.24***	
Step 3: Interaction		.00		.00
Indirect exposure ×Resilience	–.01		–.05	
Step 1: Control variables		.05		.05
Age	.07		.15***	
Gender^a^	–.22***		–.18***	
Step 2: Independentvariables		.05		.07
Worry about others	.18***		.15***	
Resilience	–.14***		–.23***	
Step 3: Interaction		.01		.01
Worry about others ×Resilience	–.08*		–.09*	
Step 1: Control variables		.05		.05
Age	.07		.15***	
Gender^a^	–.22***		–.18***	
Step 2: Independentvariables		.02		.06
House damage	.13***		.08*	
Resilience	–.13***		–.23***	
Step 3: Interaction		.00		.00
House damage ×Resilience	–.04		–.04	

*p<.05. ***p<.001.

a for adolescent participants’ gender, 0 =  female, 1 =  male.

**Figure 1:**
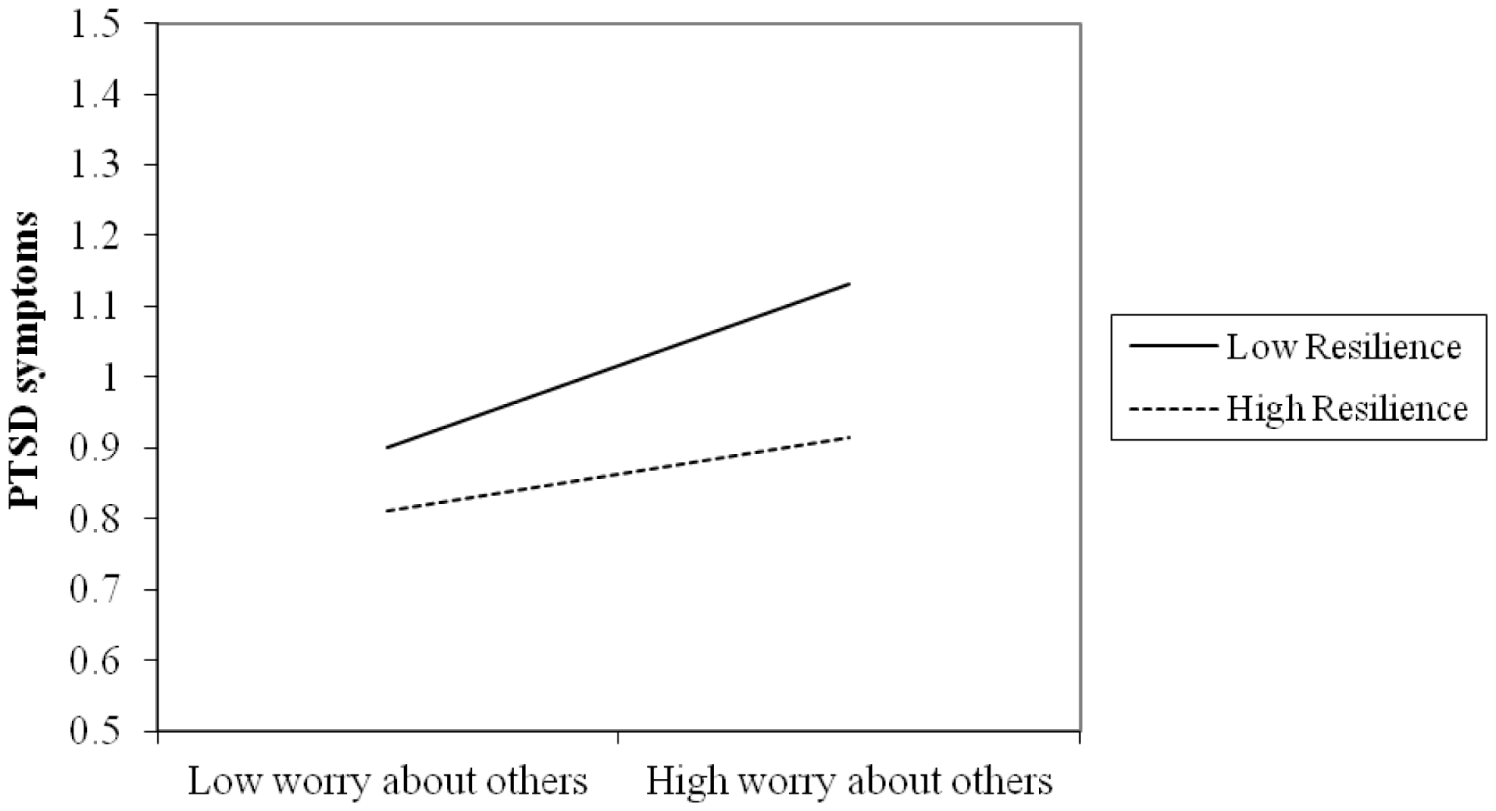
PTSD symptoms: Worry about others × Trait resilience. This figure revealed the moderation of trait resilience in the association between subjective experience (i.e., worry about others) and PTSD symptoms. For participants with low level of trait resilience, worry about others was significantly and positively associated with individual PTSD symptoms. In contrast, participants with high level of trait resilience evidenced little variation in PTSD symptoms as a function of worry about others.

**Figure 2:**
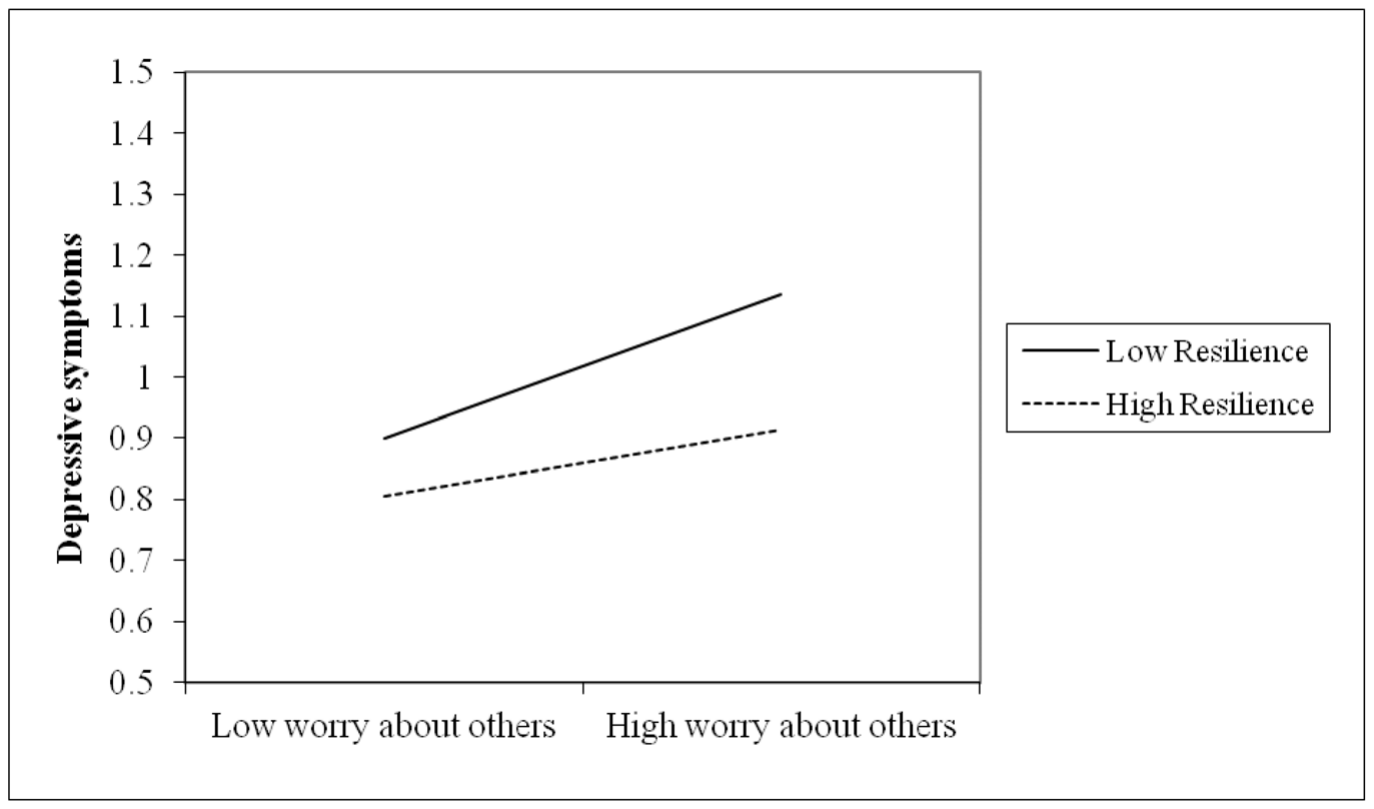
Depressive symptoms: Worry about others × Trait resilience. This figure revealed the moderation of trait resilience in the association between subjective experience (i.e., worry about others) and depressive symptoms. For participants with low level of trait resilience, worry about others was significantly and positively associated with individual depressive symptoms. In contrast, participants with high level of trait resilience evidenced little variation in depressive symptoms as a function of worry about others.

To further examine the significant interaction terms [52], we graphed PTSD and depressive symptoms for participants who were either 1 standard deviation above or below the mean with respect to worry about others as well as either 1 standard deviation above or below the mean on trait resilience. As can be seen in Figure 1 and Figure 2, for participants with a low level of trait resilience, worry about others was positively related to individual PTSD and depressive symptoms. In contrast, participants with high level of trait resilience evidenced little variation in PTSD and depressive symptoms as a function of worry about others. Using the simple slope syntax [53], we further tested whether the simple slopes of the interactions were significantly different than zero. For participants 1 standard deviation below the mean on trait resilience, the slope from low to high worry about others was associated with a positive and significant increase in PTSD (β = 23, *p*<.001) and depressive symptoms (β = 20, *p*<.001). By contrast, for participants 1 standard deviation above the mean on trait resilience, the slope from low to high worry about others against PTSD (β = .11, *p*<.05) and depressive symptoms (β = .14, *p*<.01) was flat.

## Discussion

In the current study, we examined the associations between trauma severity, trait resilience and PTSD as well as depressive symptoms among adolescent survivors of the Wenchuan earthquake. Results showed that after controlling the effect of age and gender, aspects of trauma severity (i.e., direct exposure, indirect exposure, worry about others, and house damage) had positive associations with the severity of PTSD and depressive symptoms, whereas trait resilience had a negative relationship with individual PTSD and depressive symptoms and moderated the relationship between subjective experience (i.e., worry about others) and PTSD as well as depressive symptoms.

Consistent with the previous studies [11]–[18], results indicated that after controlling the effect of age and gender, several objective elements of adolescents’ disaster experience (i.e., one’s injuries/trapped, house damage, and close one’s exposure to earthquake-related stressors) were significantly and positively associated with the severity of PTSD and depressive symptoms. The findings provide empirical support for the “dose response effect” [54], [55]. That is, greater exposures are usually associated with greater PTSD and depressive symptoms.

Furthermore, results showed that even after omitting the effect of age and gender, one subjective element (i.e., worry about others) of adolescents’ earthquake experience was significantly and negatively associated with PTSD and depressive symptoms. It is consistent with the previous studies [13], [19], [20]. One potential explanation is that subjective experiences such as worry about others at time of disaster have been found to have an inverse relationship with adaptive coping abilities [56], which are essential to resiliency and healthy outcomes following a trauma [57]. Thus, this finding suggests that subjective elements of disaster experience may play important role in determining an adolescent’s post-disaster response.

Additionally, results showed that trait resilience, measured by the CD-RISC total score, was negatively associated with PTSD and depressive symptoms among adolescent exposure to the Wenchuan earthquake. Put differently, compared to those individuals with low trait resilience, individuals with high trait resilience exhibited fewer symptoms of PTSD and depression. The current findings add to a growing body of evidence on the salubrious effect of trait resilience [28], [58], [59]. One potential explanation is that as the capacity of coping successfully with adversity [28], [60], higher trait resilience is usually associated with more positive emotion [61], [62] and increased emotional flexibility [63], which may help to reduce the likelihood of PTSD and depression and protect from psychological breakdown in the aftermath of a traumatic event [64], [65]. Thus, the present findings suggest that individual difference in trait resilience appears useful to understand effective adaptation to extreme adversity.

More importantly, our results showed that trait resilience moderated the relationship between subjective aspect of trauma severity (i.e., worry about others) and symptoms of PTSD and depression. While those individuals with a high level of trait resilience did show a significant increase in PTSD and depressive symptoms as a function of worry about others, it was still considerably less of an increase than individuals with low level of resilience. The small but significant effects of worry about others on PTSD and depressive symptoms in high-resilient adolescents point to an attenuating rather than eliminating effect of resilience on the stress-outcome relationship. The finding is consistent with Pinquart’s (2009) study of 1221 German adolescents, which found that dispositional resilience played important role in buffering the effect of the frequency of daily hassles on concurrent levels of symptoms distress [33]. Thus, the findings of the current study suggest that characteristics of resilience (e.g., tenacity, personal strength, and optimism) serve as a “buffer” against perceptions of stress and psychopathology.

Several limitations of the current study need to be mentioned. First, participants in the current study were a convenience sample from secondary schools in the counties of Wenchuan and Maoxian, the two areas most severely affected by the Wenchuan earthquake. Thus, the extent that findings of the present study may be generalized to adolescents exposed to other traumas (e.g., serious motor-vehicle accidents, sexual abuse) remains unclear. Second, all variables were measured by self-report questionnaires. Thus, the associations between trauma severity, trait resilience, and PTSD symptoms might have been inflated due to shared-method variance [66]. Additionally, because the first assessment did not take place until one year after the earthquake, it is not clear how much recollection and appraisal of the event has been distorted and whether trait resilience had been influenced by other confounding variables (e.g., social support) during this time period [61]. Finally, the cross-sectional design of this study limited its ability to draw causal inference regarding the observed relationships.

Notwithstanding these limitations, the findings of the current study have some important implications for psychological service providers to understand adolescents exposed to disaster and to provide them with an effective intervention. Our findings suggest that several aspects (i.e., direct exposure, indirect exposure, worry about others, and house damage) of earthquake experiences may be important risk factors for the development and maintenance of PTSD and depression. Moreover, the findings that trait resilience protects against PTSD and depressive symptoms and buffer the effect of subjective experience (i.e., worry about others) on PTSD and depressive symptoms, suggest that psychological service providers/school psychologists may alleviate symptoms of PTSD and depression by means of enhancing the amenable characteristics of resilience in adolescent exposed to adversity, risk, or trauma. For example, a preliminary study of 39 veterans with a variety of traumatic exposure suggested that a PTSD intervention designed to enhance resilience capacities (e.g., awareness of positive emotions) may yield broad benefits, including alleviation of symptoms and improved positive emotional and cognitive function [67]. In the long run, when considering resilience as a personality trait, it suggests that school psychologists should do much to prompt resilience in the early child life, and to encourage the development of factors associated with greater resilience in high-risk child [68].
